# Oncosuppressive functions of PIDD1 in response to centrosome amplification

**DOI:** 10.1038/s41419-021-03467-4

**Published:** 2021-02-11

**Authors:** Ilio Vitale, Gwenola Manic, Lorenzo Galluzzi

**Affiliations:** 1 IIGM—Italian Institute for Genomic Medicine, c/o IRCSS Candiolo, Torino, Italy; 2 Candiolo Cancer Institute, FPO-IRCCS, Candiolo, Italy; 3 Department of Radiation Oncology, Weill Cornell Medical College, New York, NY USA; 4 Sandra and Edward Meyer Cancer Center, New York, NY USA; 5 Caryl and Israel Englander Institute for Precision Medicine, New York, NY USA

**Keywords:** Tumour heterogeneity, Cell division

The centrosome is an intracellular organelle composed of two orthogonal centrioles surrounded by a matrix of proteins known as the pericentriolar material^1^. Besides constituting the primary microtubule organizing center of the cell, centrosomes have a key role in processes as diverse as mitosis, in which mature centrosomes orchestrate the formation of mitotic spindle, and ciliogenesis, in which centrioles act as basal bodies^1^. Centrosomes also operate as scaffolds for the recruitment (and possibly the activation) of proteins that control genomic stability, cell cycle progression, and other cellular processes^2^.

Centrosomes and DNA duplication are synchronized by the same regulatory machinery, which depends on cyclin-dependent kinase (CDK)–cyclin complexes. Specifically, CDK–cyclin complexes ensure mitotic spindle bipolarity and the faithful partition of both replicated DNA and centrosomes into daughter cells. Abundant preclinical and clinical evidence suggests that centrosome deregulation is associated with pathological conditions including cancer and microcephaly^3^. In particular, supernumerary centrosomes are frequently found in malignant tissues including precancerous lesions and reportedly promote oncogenesis as well as disease progression^4,5^. Supernumerary centrosomes can arise from centriole overduplication due to polo like kinase 4 (PLK4) deregulation (not necessarily associated with changes in cell ploidy) or whole-genome duplication upon cytokinesis abortion or cell fusion (linked to increased cell ploidy)^1^.

Irrespective of source, supernumerary centrosomes mediate oncogenic effects that have been linked to chromosome mis-segregation consequent to the assembly of multipolar and/or otherwise defective bipolar spindles^1^. In the latter setting, the clustering of extra centrosomes in two dominant mitotic spindle poles favors the mis-attachment of chromosomes to microtubules, potentially leading to aneuploidy^6^. Alongside, extra centrosomes favor metastatic dissemination by perturbing cell adhesion due to increased Rac family small GTPase 1 (RAC1) activity^7^ or by promoting the release of chemotactic factors such as interleukin-8 as a consequence of oxidative stress^8^.

A variety of oncosuppressive mechanisms orchestrated by tumor protein p53 (TP53, best known as p53) limits the proliferation or survival of cells presenting extra centrosomes, including cell cycle arrest, cellular senescence, regulated cell death, and (at least in the case of whole-genome duplication) immunosurveillance mediated by effector cytotoxic T cells^9^. CASP2 is a central player in p53 activation by extra centrosomes. In turn, CASP2 activation relies on the co-called “PIDDosome”, a supramolecular complex consisting of p53-induced death domain protein 1 (PIDD1) as well as CASP2 and RIPK1 domain containing adaptor with death domain (CRADD, best known as RAIDD) that ultimately promotes the CASP2-mediated inactivation of the p53 inhibitor MDM2 proto-oncogene (MDM2)^10^.

Two recent articles have shed light on the mechanisms whereby the PIDDosome promotes cell cycle arrest in cells with supernumerary centrosomes, revealing the implication of a CASP2-activating signal that emerges during interphase from clustered centrosomes following the recruitment of PIDD1 on distal centriole appendages^11,12^. In a first study, Evans and colleagues performed a genome-wide CRISPR-Cas9 knockout screen on immortalized retinal pigment epithelial cells (hTERT RPE-1 cells) that were engineered for the inducible overexpression of PLK4—to promote centriole overduplication—and constitutive depletion of ubiquitin-specific peptidase 28 (USP28) and tripartite motif containing 37 (TRIM37)—to silence the pathways normally activated by centrosome depletion. This screen led to the identification of 30 genes involved in the proliferation arrest of hTERT RPE-1 cells with supernumerary centrosomes, the vast majority of which (23) previously linked to centrosome functions^12^. These genes encode for well-known PIDDosome components or signal transducers: PIDD1, CRADD, CASP2, p53, and cyclin-dependent kinase inhibitor 1A (CDKN1A, best known as p21), as well as for four proteins not yet connected to PIDDosome signaling: centrosomal protein 20 (CEP20, best known as FOPNL), C2 domain containing three centriole elongation regulator (C2CD3), sodium channel and clathrin linker 1 (SCLT1), and ankyrin repeat domain 26 (ANKRD26). The capability of these proteins to arrest the proliferation of PLK4-overexpressing cells was confirmed by competition assays.

Next, Evans and colleagues focused on the impact of ANKRD26 on PIDDosome activation. First, through 3D stochastic optical reconstruction microscopy, they demonstrated that the absence of ANKRD26 limits the recruitment of PIDD1 at the distal appendages of centrioles, an effect that was not linked with defects in centrosome structure and function^12^. Limited centrosome recruitment resulted in decreased PIDDosome activation, as demonstrated by reduced CASP2 activation and p21 upregulation in PLK4-overexpressing, ANKRD26-deficient RPE-1 cells. In subsequent experiments based on *ANKRD26-* or *PIDD1*-competent vs. incompetent RPE-1 cells and a panel of full-length proteins, mutants lacking specific domains or amino acids, as well as non-cleavable mutants, the authors demonstrated that PIDD1 recruitment at centrioles occurs through the interaction between the acid region of ANKRD26 and the UPA domain of PIDD1 C-terminus (PIDD1-CC, derived from PIDD1 autoproteolysis)^12^. Importantly, in the PLK4-inducible setting, such interaction was required for PIDDosome activation and cell cycle arrest. Finally, by analyzing a panel of 20 human tumors of different origins, Evans and collaborators identified a recurrent mutation in *ANKRD26* that negatively influences the ability of ANKRD26 and PIDD1 to associate with centrosomes *in cellula*, hence improving the survival of cells with supernumerary centrosomes^12^.

In an independent study, Burigotto et al.^11^ performed stimulated emission depletion microscopy and a PIDD1 fragment-based yeast-two-hybrid screen on hTERT RPE-1 and/or lung cancer cells, providing additional evidence that PIDD1 interacts with ANKRD26 at the distal appendages of mature centrosomes. In following epistatic analyses, these authors showed that PIDD1 binds centrioles through a process that involves the sequential recruitment of centrosomal protein 83 (CEP83), SCLT1, and ANKRD26 (ref. ^11^). Complementation studies in ANKRD26-deficient RPE-1 cells identified ANKRD26 amino acids 911–1181 (dubbed PMID) as the interaction domain for PIDD1 to localize to centrosomes. Moreover, MDM2 cleavage evaluation by immunoblotting suggested defective PIDDosome activation in RPE-1 cells deficient for CEP83, SCLT1, ANKRD26 or PIDD1 upon exposure to the tetraplodizing agent dihydrocytochalasin B^11^. In re-expression studies based on human non-small cell lung carcinoma *PIDD1*^*−/−*^ A549 cells, Burigotto et al.^11^ demonstrated that only full-length PIDD1 localizes to centrosome and that, despite occurring independently from centrosome localization, PIDD1 autoproteolysis is strictly required for PIDDosome activation. Finally, live videomicroscopy and fluorescence recovery after photobleaching analyses demonstrated a rapid exchange of the cytoplasmic and centrosomal pool of PIDD1 as well as an increase in the concentration of PIDD1-CC in response to supernumerary centrosome clustering during interphase. Accordingly, an experimental strategy to limit centrosome clustering based on nocodazole-driven microtubule depolymerization in conditions of mitotic slippage resulted in decreased PIDDosome activation and cell cycle arrest. Finally, the DNA damaging agent camptothecin was shown to activate the PIDDosome via a mechanism independent on centrosome amplification but dependent on PIDD1 localization to, and activation at, the centrosome^11^.

**Fig. 1:**
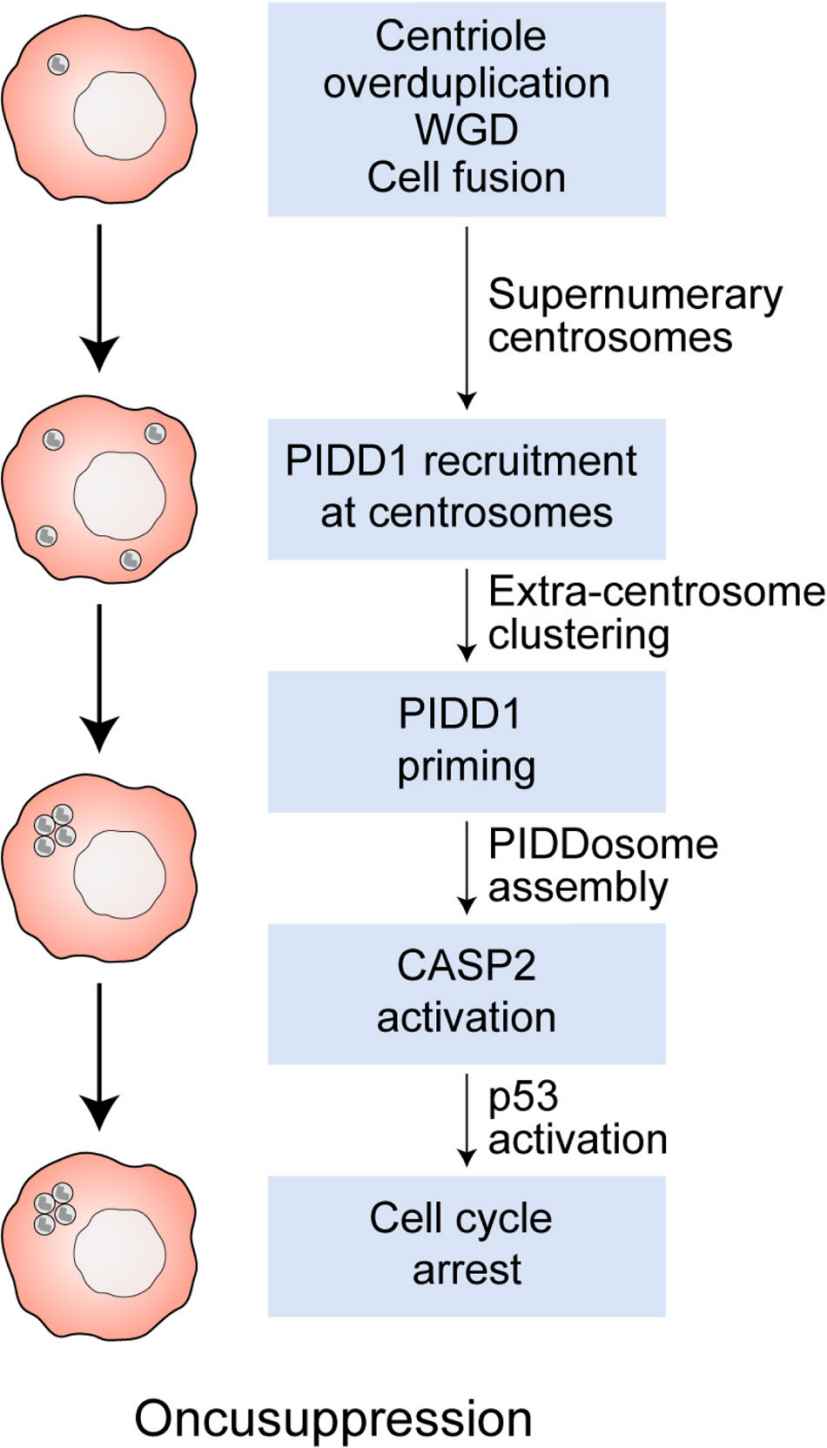
Extra centrosomes halt cell cycle progression by a PIDD1-dependent mechanism. The cell cycle of cancer cells acquiring supernumerary centrosomes is arrested by a mechanism involving the recruitment of PIDD1 to the distal appendages of mature centrosomes via ANKRD26, followed by centrosome clustering, and PIDDosome signaling. This results in CASP2 activation, MDM2 degradation, and the initiation of a p53-dependent cascade arresting cell cycle progression.

In conclusion, these two studies uncovered a novel role of PIDD1 as a sensor of centrosome amplification that involves (1) PIDD1 recruitment to centrioles, (2) centrosomes clustering during interphase, (3) PIDD1 autoproteolysis, (4) CASP2 activation and consequent MDM2 degradation, and (5) p53-dependent, p21-executed cell cycle arrest (Fig. 1). Preliminary findings indicate that this cascade can suppress tumorigenesis and may be involved in other pathological conditions such as the autosomal dominant thrombocytopenia caused by platelet depletion.

Given the role of CASP2 in mitotic catastrophe^9^ and mitochondrial outer membrane permeabilization, it will be important to explore whether PIDD1 also promotes the demise of cells with supernumerary centrosomes. Alongside, future studies should explore whether and how extra centrosomes influence (i) the DNA damage response (DDR), as several DDR players associate with centrosomes and are deregulated in human cancers, and (ii) anti-tumor immunity, as the stress response initiated by supernumerary centrosomes may promote the release of damage-associated molecular patterns^13^ for the recruitment/activation of immune effector cells. In this context, understanding the mechanisms harnessed by cells with supernumerary centrosomes to bypass CASP2-mediated oncosuppression beyond p53, CASP2, and ANKRD26 defects^12,14^ will shed additional light on the capacity of malignant cells to tolerate aneuploidy and potentially guide the design of novel therapeutic strategies against cancer.
